# Risk factors of unmet needs among women with breast cancer in the post‐treatment phase

**DOI:** 10.1002/pon.5299

**Published:** 2019-12-16

**Authors:** Deborah N. N. Lo‐Fo‐Wong, Hanneke C. J. M. de Haes, Neil K. Aaronson, Doris L. van Abbema, Mathilda D. den Boer, Marjan van Hezewijk, Marcelle Immink, Ad A. Kaptein, Marian B. E. Menke‐Pluijmers, Anna K. L. Reyners, Nicola S. Russell, Manon Schriek, Sieta Sijtsema, Geertjan van Tienhoven, Mathilde G. E. Verdam, Mirjam A. G. Sprangers

**Affiliations:** ^1^ Academic Medical Center, Cancer Center Amsterdam Amsterdam University Medical Centers Amsterdam The Netherlands; ^2^ Antoni van Leeuwenhoek Hospital The Netherlands Cancer Institute Amsterdam The Netherlands; ^3^ GROW – School for Oncology and Developmental Biology Maastricht University Medical Center Maastricht The Netherlands; ^4^ Erasmus MC Cancer Institute Rotterdam The Netherlands; ^5^ Leiden University Medical Center Leiden The Netherlands; ^6^ Reinier de Graaf Hospital Delft The Netherlands; ^7^ Albert Schweitzer Hospital Dordrecht The Netherlands; ^8^ University of Groningen University Medical Center Groningen Groningen The Netherlands; ^9^ St. Elisabeth Hospital Tilburg The Netherlands; ^10^ University Medical Center Utrecht Utrecht The Netherlands; ^11^ Present address: Erasmus University Medical Center Rotterdam the Netherlands; ^12^ Present address: Treant Zorggroep, Scheper Hospital Emmen the Netherlands; ^13^ Present address: Radiotherapy Group Arnhem the Netherlands; ^14^ Present address: Isala Clinics Zwolle the Netherlands

**Keywords:** cancer, distress, needs assessment, oncology, women with breast cancer

## Abstract

**Objective:**

Unmet health care needs require additional care resources to achieve optimal patient well‐being. In this nationwide study we examined associations between a number of risk factors and unmet needs after treatment among women with breast cancer, while taking into account their health care practices. We expected that more care use would be associated with lower levels of unmet needs.

**Methods:**

A multicenter, prospective, observational design was employed. Women with primary breast cancer completed questionnaires 6 and 15 months post‐diagnosis. Medical data were retrieved from medical records. Direct and indirect associations between sociodemographic and clinical risk factors, distress, care use, and unmet needs were investigated with structural equation modeling.

**Results:**

Seven hundred forty‐six participants completed both questionnaires (response rate 73.7%). The care services received were not negatively associated with the reported levels of unmet needs after treatment. Comorbidity was associated with higher physical and daily living needs. Higher age was associated with higher health system‐related and informational needs. Having had chemotherapy and a mastectomy were associated with higher sexuality needs and breast cancer‐specific issues, respectively. A higher level of distress was associated with higher levels of unmet need in all domains.

**Conclusions:**

Clinicians may use these results to timely identify which women are at risk of developing specific unmet needs after treatment. Evidence‐based, cost‐effective (online) interventions that target distress, the most influential risk factor, should be further implemented and disseminated among patients and clinicians.

## 1 BACKGROUND

Breast cancer is the most frequently diagnosed cancer among women worldwide.^1^ Thanks to the introduction of early detection programs and increasingly successful treatments, the number of breast cancer survivors keeps rising. Yet, this positive development entails an increasing number of survivors with disease‐ and treatment‐related problems. Many report fatigue, psychological distress, poor physical fitness, motion restriction, lymphedema, sleep problems, cognitive problems, and menopausal symptoms.^2^, ^3^, ^4^, ^5^

Many women with breast cancer receive medical, paramedical, psychosocial, or complementary care to cope with these problems, up to more than 10 years after diagnosis.^6^ For most women, the received resources are sufficient.^7^ However, a considerable proportion of women with breast cancer express an unmet need for support, indicating that they would like additional help. The prevalence of specific needs may reach up to 70%. The highest needs are generally found in the psychological and health system‐related and informational domain, with fear of the cancer spreading or recurring being the most prevalent.^8^

To ensure that these needs are adequately addressed, clinicians might benefit from knowing which women with breast cancer are most at risk of developing unmet needs. A considerable number of studies addressed this topic. A systematic review found that, for instance, younger women, with advanced stage breast cancer, treated with chemotherapy, or those who experience a higher level of distress, report increased unmet needs. Risk factors may differ between domains. For example, women with a higher level of education report greater unmet need in the sexuality, and/or health system‐related and informational domains, while women with a lower level of education generally report greater unmet need in the psychological and/or patient care and support domains.^8^

These insights show promise in identifying the women with breast cancer most in need for additional support. However, more research is warranted. First, most studies employed cross‐sectional designs to address risk factors of unmet need domains during cancer treatment, when the *levels* of unmet needs are highest. Far less studies examined risk factors of need domains shortly after treatment, when a greater *number* of patients experience unmet needs.^9^ Patients' need for support may rise as they may miss regular contact with health care professionals and/or experience treatment‐induced side effects.^10^ Therefore, more prospective studies regarding risk factors of specific unmet need domains in the post‐treatment phase are required.^11^

Furthermore, health care needs, by definition, refer to problems that require an action or additional care resources to achieve optimal well‐being.^8^, ^9^, ^12^ This implies that received care resources and one's level of unmet need may be associated.^13^, ^14^ Yet, to the best of our knowledge, the extent to which different types of received health care, such as medical, paramedical, or psychosocial care, influence the relation between risk factors and specific unmet need domains, has not been investigated.

The current study aims to extend existing insights by examining associations between risk factors and unmet needs of women with breast cancer post‐treatment, while taking into account varying types of care use. We hypothesized that higher levels of health care use are associated with lower levels of remaining unmet needs, thus that care use helps fulfill existing needs.

Based on the literature, we included age, educational level, cancer stage, types of treatment, and distress as sociodemographic, clinical, and psychosocial risk factors.^8^ We additionally included type of care insurance and comorbidity as possible risk factors that deserve more research attention.^14^, ^15^ Patients may refrain from physical or psychological treatment if their insurance does not fully cover the costs. Consequently, they may experience higher levels of unmet need in the physical and daily living and psychological domains. Having one or more comorbid disorders may especially influence unmet need in the physical and daily living domain. Finally, we included previous psychosocial treatment as a potential risk factor. Breast cancer patients with a history of mental illness are at higher risk of developing cancer‐related distress.^16^ If not adequately addressed, they may experience higher levels of psychological need over time.

Most of the included sociodemographic and clinical factors are also known risk factors for a higher level of distress.^17^ As such, distress might be a more proximal risk factor, and possibly a mediating factor between the other risk factors and patients' levels of unmet needs. Therefore, we further hypothesized that the included sociodemographic and clinical factors influence unmet needs directly and indirectly through distress.

## 2 METHODS

### 2.1 Participants and procedure

Women with primary breast cancer diagnosed up to 6 months earlier in one of nine hospitals in the Netherlands (ie, six academic hospitals, two community hospitals, and one comprehensive cancer center) were eligible for the study, regardless of type of treatment. Patients were excluded if they were not literate in Dutch, younger than 18 years, and/or had a prognosis of 3 months or less.

Eligible patients were identified by their oncologist, cancer nurse, or nurse practitioner during a hospital visit. The clinician informed the patient about the study and asked whether she would consider participation. Subsequently, the investigator invited interested patients to participate by telephone or e‐mail. Participating centers could exclude patients who were already participating in other studies. A more detailed description of the study procedure has previously been published.^7^, ^18^

Our aim was to include at least 900 participants, a sufficient number for testing multiple correlations, given the number of predictors. This number would suffice even when domain scores would be included separately, and taking into account a drop‐out rate of 20%.^19^, ^20^

### 2.2 Design

The study had a multicenter, prospective, observational design. Participants completed a self‐report questionnaire at 6 months (time window 5 to 7 months) and 15 months (time window 14 to 16 months) post‐diagnosis. Medical data were retrieved from medical records. As the study was observational in nature, it did not require formal review according to the institutional review boards of participating hospitals, in accordance with Dutch legal regulations.

Sociodemographic factors were assessed at 6 months post‐diagnosis. Distress, health care use and needs were assessed at 6 and 15 months post‐diagnosis. As we were interested in identifying risk factors of unmet needs over time, after adjusting for received care, we included the data on sociodemographic, clinical, and psychosocial risk factors from the 6 months post‐diagnosis assessment, and the data on care use and needs from the 15 months post‐diagnosis assessment.

### 2.3 Sociodemographic and clinical factors

Age at diagnosis, educational level (8 response categories, including the option “other,” see the legend of Table 2), type of health insurance (5 response categories: no health insurance, basic package, basic package and additional package without dental insurance, basic package and dental insurance, basic package with additional and dental insurance), number of comorbid conditions (17 response categories, including the options “none” and “other”),^21^ and previous use of psychosocial services (yes/no) were assessed through self‐report. Cancer stage (via pTNM‐classification)^22^ and types of treatment were retrieved from medical records.

### 2.4 Distress

Psychosocial distress was assessed with the validated Dutch version of the single item Distress Thermometer.^23^, ^24^ The Thermometer is a visual analog scale, that measures the level of distress in the past week (score 0 “no distress at all” to 10 “extreme distress”).

### 2.5 Health care use

Health care use was assessed with a 24‐item questionnaire.^7^, ^18^, ^21^ The questionnaire measures how often in the past 3 months patients used specific types of medical (eg, visits to a surgeon), psychosocial (eg, visits to a psychologist), paramedical (eg, visits to a physical therapist), and supplementary care services (eg, use of paid child care) (response categories: 0, 1, 2, 3, 4, 5, more than 5 times). The legend of Table 2 provides an overview of the included services. We calculated a sum score for each type of care.

### 2.6 Health care needs

Health care needs were assessed with the 34‐item Supportive Care Needs Survey (SCNS‐SF34),^25^ and the 8‐item SCNS breast cancer‐specific module.^26^ The SCNS measures patients' perceived needs over the past month in the psychological, health system‐related and informational, physical and daily living, patient care and support, and sexuality domain (five response categories: no need, met need, some need, moderate need, high need). The additional module addresses breast cancer‐specific needs, for example, related to experiencing lymphedema.

We calculated a Likert sum score for each domain, excluding the category “met need,” such that a higher domain score indicated a higher level of unmet need for help (range 0 “no need” to 3 “high need”). This was an adaptation of the standard scoring procedure. Cronbach's alpha coefficient in our study ranged from 0.86 to 0.96 for the SCNS‐SF34 subscales and was 0.81 for the breast cancer‐specific module. Finally, we transformed the scores to a scale ranging from 0 to 100 in order to facilitate comparisons across scores.

### 2.7 Data analyses

Associations between the included risk factors, care use, and unmet needs over time were examined with structural equation modeling (SEM).^27^ SEM has the ability to include multiple independent and dependent variables in one model, thus enabling simultaneous analysis of all hypothesized associations. Missing values ranged from 0% for age and the treatment‐related variables to 9.5% for the unmet need score in the patient care and support domain. The full information maximum likelihood estimation method^28^ was used to take missing data into account.^29^ SEs were corrected for deviations from normality by use of the Huber‐White estimator.^30^

Our model included direct effects of all sociodemographic and clinical factors on level of distress, care use, and needs factors and of all care use factors on all care need variables. Residual covariances were allowed between health care use or health care need variables. The resulting specified model was a saturated model with zero degrees of freedom.

The reported standardized parameters (ß's) can be interpreted as effect size indices. Values of 0.10, 0.30, and 0.50 were considered to indicate small, medium, and large effect sizes for categorical variables, and values of 0.20, 0.50, and 0.80 for continuous variables. For the reported R‐squares—the percentages of explained variance in distress, care use, and needs—values of 2%, 13%, and 26% were considered to reflect small, medium, and large effect sizes, respectively.^31^

Descriptive analyses were performed with SPSS version 22. For SEM, the lavaan package in the R software system for statistical computing was used.^32^

## 3 RESULTS

### 3.1 Sample

**Figure 1:**
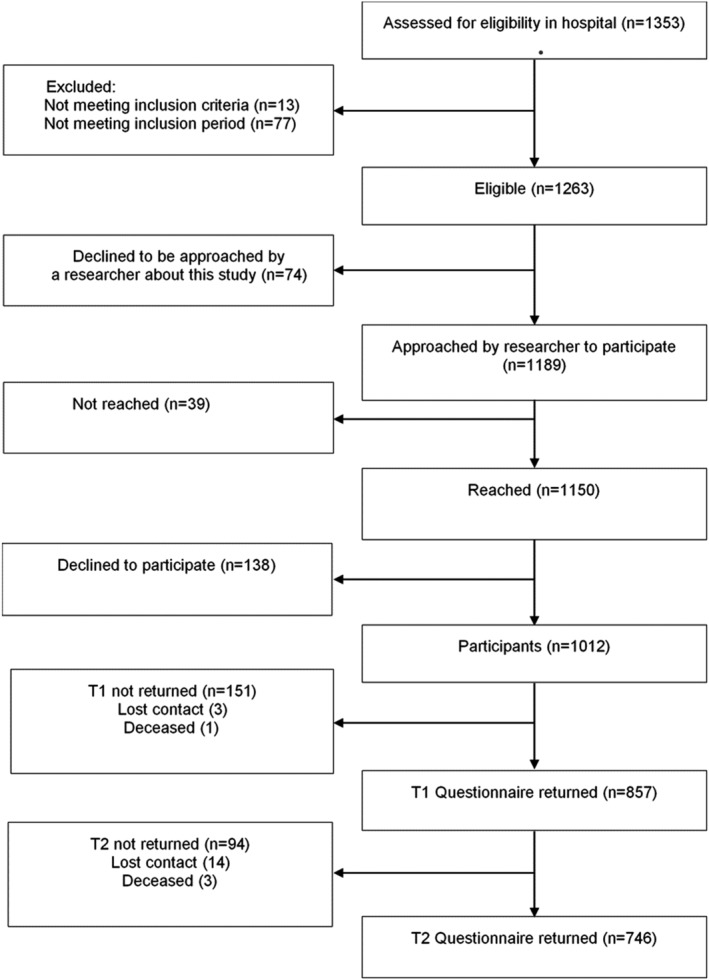
Flowchart

Of 1353 women with breast cancer assessed, 1263 women were eligible, and 1012 agreed to participate. The analyses for this study included the 746 women who completed both questionnaires (73.7% of participants) (Figure 1). Most women were diagnosed with stage 1 or 2 invasive breast cancer, and were treated with lumpectomy and radiotherapy. Over 60% had one or more comorbid conditions (Table 1).

**Table 1:** Sample characteristics (n = 746)

Characteristics	Total sample^a^
Sociodemographic factors	
Age at diagnosis (median, range)	58 (24‐83)
Educational level (n, %)^b^	
Low	345 (46.3)
Intermediate	185 (24.8)
High	215 (28.9)
Health insurance	
No health insurance	2 (0.3)
Basic package	83 (11.4)
Basic and additional package	645 (88.4)
Clinical factors	
Cancer stage at diagnosis (n, %)	
TIS: carcinoma in situ	103 (13.9)
Invasive early stage (T1/T2)	620 (83.6)
Invasive late stage (T3/T4)	19 (2.6)
Type of surgery (n, %)	
Lumpectomy	630 (84.5)
Mastectomy	105 (14.1)
Lumpectomy and mastectomy	9 (1.2)
No lumpectomy or mastectomy	2 (0.3)
Radio and/or chemotherapy (n, %)	
Radiotherapy only	470 (63.0)
Chemotherapy only	24 (3.2)
Radio‐ and chemotherapy	198 (26.5)
No radio‐ or chemotherapy	54 (7.2)
Other types of treatment (n, %)	
Hormonal therapy (yes)	258 (34.6)
Immunotherapy (yes)	32 (4.3)
Comorbid conditions (n, %; yes)	457 (62.2)
Previous use of psychosocial services (n, %; yes)	167 (22.5)

a Presented percentages are valid percentages, missing values excluded.

b Educational level was categorized as low (no education, elementary school, low level vocational education, or intermediate level high school), intermediate (intermediate level vocational education, or high level high school), and high (high level vocational education, or college or university).

Participants did not differ significantly in age (groups based on median split, *P* > .10), cancer stage (chi‐square, *P* > .10), or distress score 6 months post‐diagnosis (*P* > .10) from nonrespondents, that is, from women who were approached by the investigator, but could not be reached or declined to participate. Participants who only completed the first questionnaire at 6 months post‐diagnosis (n = 111) did not differ significantly in age (chi‐square, *P* > .10), cancer stage (chi‐square, *P* > .10), or distress score (*t* tests, *P* > .10) from those who completed both questionnaires (n = 746).

### 3.2 Risk factors of distress

Patients reported on average a distress level of 3.93 (SD = 2.67) at 6 months post‐diagnosis. Younger age (ß = −.18), having had chemotherapy (ß = .43), comorbidity (ß = .17), and psychosocial treatment before the breast cancer diagnosis (ß = .29) were found to be associated with a higher level of distress (*P* < .05). The predictors together explained 12.3% of variance, reflecting a small effect size (Table 2).

**Table 2:** Predictors of distress, health care use, and unmet needs at 15 months post‐diagnosis*

Factor^a^, ^c^	ß Est.^b^	SE*	Z‐value	*P*(>|z|)	*R* ^2^
*Level of distress*					12.30%
Age	−.177	0.044	−4.028	**.000**	
Educational level 1	.015	0.091	0.161	.872	
Educational level 2	−.072	0.086	−0.838	.402	
Type of health insurance	.043	0.111	0.390	.696	
Cancer stage	−.072	0.113	−0.637	.524	
Mastectomy	−.003	0.146	−0.021	.983	
Chemotherapy	.431	0.098	4.417	**.000**	
Radiotherapy	−.073	0.161	−0.455	.649	
Hormonal therapy	.061	0.085	0.717	.473	
Comorbidity	.174	0.077	2.255	**.024**	
Psychosocial treatment before diagnosis	.290	0.083	3.491	**.000**	
*Unmet needs*					
*Physical and daily living needs*					24.10%
Age	−.023	0.040	−0.574	.566	
Educational level 1	.087	0.087	1.000	.317	
Educational level 2	.042	0.080	0.530	.596	
Type of health insurance	−.108	0.103	−1.050	.294	
Cancer stage	.164	0.088	1.860	.063	
Mastectomy	.252	0.139	1.812	.070	
Chemotherapy	−.056	0.102	−0.556	.578	
Radiotherapy	.174	0.159	1.097	.273	
Hormonal therapy	−.104	0.082	−1.268	.205	
Comorbidity	.146	0.073	1.999	**.046**	
Psychosocial treatment before diagnosis	.174	0.090	1.934	.053	
Level of distress	.372	0.041	9.126	**.000**	
Medical care use	.083	0.038	2.162	**.031**	
Psychosocial care use	.095	0.048	1.959	**.050**	
Paramedical care use	−.048	0.031	−1.526	.127	
Supplementary service use	.086	0.062	1.393	.164	
*Patient care and support needs*					8.00%
Age	−.033	0.051	−0.643	.520	
Educational level 1	.030	0.090	0.334	.739	
Educational level 2	.060	0.087	0.689	.491	
Type of health insurance	−.092	0.123	−0.744	.457	
Cancer stage	.138	0.084	1.635	.102	
Mastectomy	.372	0.217	1.718	.086	
Chemotherapy	.124	0.112	1.102	.270	
Radiotherapy	.221	0.214	1.033	.302	
Hormonal therapy	−.140	0.091	−1.535	.125	
Comorbidity	.063	0.075	0.846	.398	
Psychosocial treatment before diagnosis	.157	0.107	1.460	.144	
Level of distress	.137	0.039	3.486	**.000**	
Medical care use	.083	0.042	1.962	**.050**	
Psychosocial care use	.073	0.041	1.804	.071	
Paramedical care use	−.069	0.034	−2.039	**.041**	
Supplementary service use	−.020	0.039	−0.514	.607	
*Psychological needs*					19.60%
Age	.004	0.042	0.098	.922	
Educational level 1	−.166	0.089	−1.870	.061	
Educational level 2	−.088	0.080	−1.096	.273	
Type of health insurance	−.078	0.114	−0.684	.494	
Cancer stage	.014	0.097	0.144	.885	
Mastectomy	.278	0.174	1.594	.111	
Chemotherapy	.048	0.110	0.434	.664	
Radiotherapy	.215	0.188	1.143	.253	
Hormonal therapy	−.126	0.087	−1.441	.150	
Comorbidity	.035	0.071	0.484	.628	
Psychosocial treatment before diagnosis	.193	0.099	1.944	.052	
Level of distress	.301	0.041	7.317	**.000**	
Medical care use	.076	0.044	1.718	.086	
Psychosocial care use	.192	0.045	4.254	**.000**	
Paramedical care use	−.049	0.049	−1.005	.315	
Supplementary service use	.003	0.045	0.074	.941	
*Sexuality needs*					11.70%
Age	−.058	0.043	−1.349	.177	
Educational level 1	−.063	0.089	−0.714	.475	
Educational level 2	.057	0.094	0.607	.544	
Type of health insurance	−.122	0.115	−1.063	.288	
Cancer stage	.015	0.094	0.154	.878	
Mastectomy	.209	0.188	1.113	.266	
Chemotherapy	.269	0.126	2.142	**.032**	
Radiotherapy	−.060	0.215	−0.281	.778	
Hormonal therapy	−.170	0.095	−1.784	.074	
Comorbidity	.015	0.074	0.201	.841	
Psychosocial treatment before diagnosis	.158	0.109	1.446	.148	
Level of distress	.130	0.038	3.434	**.001**	
Medical care use	.027	0.039	0.694	.488	
Psychosocial care use	.148	0.053	2.772	**.006**	
Paramedical care use	−.032	0.039	−0.821	.412	
Supplementary service use	−.006	0.046	−0.131	.896	
*System‐related and informational needs*					9.10%
Age	.111	0.046	2.418	**.016**	
Educational level 1	−.069	0.097	−0.709	.478	
Educational level 2	−.121	0.086	−1.406	.160	
Type of health insurance	−.222	0.136	−1.630	.103	
Cancer stage	.026	0.107	0.248	.805	
Mastectomy	.256	0.177	1.443	.149	
Chemotherapy	.131	0.117	1.126	.260	
Radiotherapy	.006	0.209	0.028	.978	
Hormonal therapy	−.100	0.095	−1.058	.290	
Comorbidity	.092	0.073	1.252	.211	
Psychosocial treatment before diagnosis	.087	0.096	0.901	.368	
Level of distress	.223	0.045	4.905	**.000**	
Medical care use	.070	0.053	1.329	.184	
Psychosocial care use	.039	0.036	1.085	.278	
Paramedical care use	−.021	0.055	−0.378	.705	
Supplementary service use	.000	0.048	0.009	.993	
*Breast cancer‐specific needs*					18.60%
Age	.050	0.043	1.147	.251	
Educational level 1	−.014	0.092	−0.148	.882	
Educational level 2	−.069	0.079	−0.878	.380	
Type of health insurance	−.188	0.119	−1.588	.112	
Cancer stage	.095	0.085	1.124	.261	
Mastectomy	.693	0.254	2.733	**.006**	
Chemotherapy	−.069	0.107	−0.647	.517	
Radiotherapy	.043	0.279	0.155	.877	
Hormonal therapy	−.056	0.091	−0.611	.541	
Comorbidity	−.075	0.079	−0.946	.344	
Psychosocial treatment before diagnosis	.082	0.093	0.882	.378	
Level of distress	.197	0.044	4.434	**.000**	
Medical care use	.199	0.066	3.021	**.003**	
Psychosocial care use	.055	0.043	1.259	.208	
Paramedical care use	−.000	0.049	−0.000	1.000	
Supplementary service use	.040	0.052	0.771	.441	

a With the exception of age, distress, health care use, and needs, all the variables were entered as dummy variables (educational level 1: low vs intermediate; educational level 2: intermediate vs high; type of insurance: basic vs basic and additional package; cancer stage: ductal carcinoma in situ vs invasive tumor; all types of treatment: no vs yes; comorbidity: no vs one or more comorbid conditions). Immunotherapy was not included as a predictor given the small percentage of participants who received this type of treatment (n = 32).

b The reported standardized parameters (ß's) can be interpreted as effect size indices. That is, for the continuous predictors age, distress, health care use, and needs, values of .20, .50, and .80 are indicative of small, medium, respectively large effect sizes. For categorical predictors values of .10, .30, and .50 are considered to indicate small, medium, large effect sizes, respectively.

c Medical care use included visits to a surgeon, radiation oncologist, medical oncologist, breast cancer nurse, anesthesiologist, general practitioner, plastic surgeon, sexologist, gynecologist, clinical geneticist, occupational physician, or lymphedema therapist. Psychosocial care use included visits to a psychologist or psychotherapist, social worker, psychiatrist, or spiritual care provider. Paramedical care use included visits to a physical therapist, dietician, or ergotherapist. Supplementary service care use included use of paid child care, home care/nurse at home, domestic help, a support group, or a group rehabilitation program.

*Printed in bold: *P* < .05.

### 3.3 Risk factors of unmet needs

Patients reported the highest level of unmet need in the physical and daily living domain (M = 13.40, SD = 20.09), followed by the psychological domain (M = 12.85, SD = 19.63), the health system‐related and informational domain (M = 11.01, SD = 20.53), and the patient care and support domain (M = 7.25, SD = 15.59) at 15 months post‐diagnosis. The lowest unmet needs were related to breast cancer‐specific (M = 6.72, SD = 13.40) and sexuality issues (M = 5.90, SD = 15.58).

A number of risk factors were found to be associated with higher levels of unmet needs, while taking into account types of care received (*P* < .05). Comorbidity (ß = .15), a higher level of distress (ß = .37), more medical care use (ß = .08), and psychosocial care use (ß = .10) were significantly associated with a higher level of unmet need in the physical and daily living domain. A higher level of distress (ß = .14), more medical care use (ß = .08), and less paramedical care use (ß = −.07) were significantly associated with a higher level of unmet need related to patient care and support. Furthermore, a higher level of distress (ß = .30) and more frequent use of psychosocial care use (ß = .19) significantly predicted a higher level of unmet needs related to psychological issues, and along with chemotherapy also significantly predicted unmet needs related to sexuality (ß = .27 for chemotherapy, ß = .13 for distress and ß = .15 for psychosocial care use). Higher age (ß = .11) and a higher level of distress (ß = .22) significantly predicted a higher level of unmet health system‐related and informational needs. Finally, having a mastectomy (ß = .69), a higher level of distress (ß = .20), and medical care use (ß = .20) significantly predicted a higher level of unmet breast cancer‐specific needs.

The risk factors explained between 8.0% of variance for unmet need in the domain of care and support and 24.1% of variance for unmet physical and daily living needs, indicating small to medium effect sizes (Table 2).

## 4 DISCUSSION

This prospective, nationwide study identified risk factors of unmet needs among women with breast cancer after treatment, while taking into account their global reports of received care practices. Of the included sociodemographic, clinical, and psychosocial risk factors, higher age, having one or more comorbid disorders, having had chemotherapy or a mastectomy, and patients' level of distress were found to be significant direct risk factors.

The data, generally, did not support our hypothesis that women's reports of *received care services* are negatively related to their unmet needs. There were even some small positive associations, for example, between medical care use and the reported level of breast cancer‐specific needs (Table 2). Perhaps the lack of negative associations between use and unmet needs domains, after controlling for women's level of distress, indicates that some health care services did not completely fulfill patients' expectations. The positive associations may reflect that some patients are prone to keep seeking and using care. Alternatively, it could be that more received care does decrease one's levels of unmet care needs, but that this may only become apparent with a longer period of follow‐up.

With regard to the *sociodemographic factors*, there is strong evidence that younger age is associated with a higher number of unmet needs after cancer treatment.^11^, ^33^ Additionally, two studies among early cancer survivors reported associations between younger age and specific unmet need domains, namely the patient care and support, and the relationship/sexuality domain.^11^ In contrast, our study showed a small but significant direct association between higher age and higher levels of unmet need in the health system‐related and informational domain. While an overall effect of age on unmet needs has been established, we conclude that this might mask varying effects on separate underlying unmet needs domains. One explanation for our finding is that older patients are more likely to experience more comorbid or physical problems in addition to their cancer diagnosis, which requires additional support and information. Also, older cancer patients may have more difficulty with processing and recalling provided information due to decreasing cognitive and sensory functions.^34^ In addition to a direct effect, we would like to highlight that age was found to be a relevant indirect risk factor of all unmet need domains, through distress.

Our study showed nonsignificant associations between patients' type of care insurance and their levels of unmet needs in varying domains. Hopefully, this is an indication that coverage is not an access barrier to receiving satisfactory care just after breast cancer treatment—at least in countries such as the Netherlands where having basic medical insurance is obligatory. The influence of patients' care insurance on their levels of unmet need will differ between care systems. We encourage researchers to further investigate the influence of access‐related risk factors on varying unmet need domains directly following treatment and in the survival phase.

Regarding the included *clinical factors*, the existing evidence on the association between cancer stage and unmet needs after treatment is inconclusive. Two studies with a cancer‐specific samples (ie, women with gynecologic or endometrial cancer) found a significant association between stage and unmet needs, as measured around 4 years after diagnosis, while one study did not. Comparable studies with breast cancer,^15^, ^33^, ^35^, ^36^, ^37^ other, or mixed cancer samples^11^ often lacked information on the cancer stage at diagnosis, and did not examine its influence on separate unmet need domains post‐treatment. Only 3% of our participants were diagnosed with cancer stage 3 or 4, which meant that we could not examine the influence of later cancer stages on women's levels of unmet needs. We were able to establish that women with carcinoma in situ do not differ in level of unmet needs after treatment from women with invasive, mostly early stage, breast cancer.

Having had chemotherapy was previously found to be the most relevant treatment‐related risk factor, and was generally associated with several unmet need domains after a breast cancer diagnosis (ie, the psychological, physical/daily living, patient care and support, and the sexuality domain). Most of these earlier studies addressed the treatment phase.^8^ Based on our results, chemotherapy may especially have longer lasting direct effects on unmet need in the sexuality domain. This finding might be breast cancer‐specific.^11^ The result is consistent with the widely held belief that both physicians and patients can be reluctant to openly discuss sexuality problems due to cancer or its treatment. In fact, we found that 10% of our sample would have liked more contact with a sexologist 12 to 15 months after diagnosis.^7^ In general, it appears that the influence of relevant clinical risk factors on unmet needs becomes more domain‐specific with time after diagnosis. Accordingly, there was a strong association between having had a mastectomy and breast‐cancer specific needs, and between comorbidity and physical and daily living needs after treatment, as hypothesized.

The most relevant risk factor of unmet needs after treatment is *distress*. A higher level of distress was found to significantly, directly influence unmet needs after breast cancer treatment across domains. This result is consistent with previous studies, regarding distress and anxiety.^33^, ^36^, ^37^ Our findings extend the literature by establishing the enduring influence of breast cancer‐related distress on specific unmet need domains over time, especially physical and daily living, psychological, and health system‐related and informational needs. Importantly, our findings indicate that distress may be an important mediator between sociodemographic and clinical risk factors (ie, age, comorbidity, psychosocial treatment before diagnosis, especially chemotherapy) and unmet needs, as previously suggested.^33^

### 4.1 Study limitations

The number of visits to care providers was assessed by self‐report. These results may be influenced by recall bias, leading to possible under‐ or overestimation of actual care practices. However, self‐assessment allowed us to also assess the use of nonmedical types of care use, which are not standardly registered in medical files. Another study limitation is that many participants were recruited from radiotherapy departments. Therefore, women with breast cancer who do not receive radiotherapy, a minority, were underrepresented in our sample. A previous study based on data from a Dutch population‐based, regional cancer registry indicated that 17% of the women with breast cancer received systemic therapy without radiotherapy.^38^ Furthermore, we were not able to gather information about women who declined to be approached for this study. Therefore, we could not determine our sample's representativeness in that regard. There was, however, no indication of a sample bias resulting from loss to follow‐up.

Strengths of our study include its multicenter, prospective, design, its nationwide character, and large sample size. We addressed a key period in the disease trajectory that warrants further investigation of risk factors in relation to survivors' unmet needs. Indeed, thanks to the large sample size, we were able to simultaneously examine multiple risk factors of separate domains of unmet needs in concurrence with varying types of health care use of women with breast cancer. To our knowledge, this is the first study to have done so.

### 4.2 Future research suggestions

To ensure appropriate and cost‐effective utilization of care services, the influence of health care use on varying domains of unmet needs post‐treatment and in the survival phase deserves further attention. We recommend employing standardized care use^39^ and needs measurements^8^ in order to allow comparisons across studies. Given the increasing number of women with breast cancer, we especially suggest further investigation of the association between psychosocial care use and unmet needs after a breast cancer diagnosis. Patients with informational or emotional needs may seek support from psychosocial providers, while their needs might be satisfied by participating in support programs, receiving written information, or using low‐cost self‐management resources. All future studies on this topic should include distress as a key risk factor, and preferably also as a mediating factor. Based on our results, we also recommend examination of types of comorbid disorders in relation to breast cancer patients' unmet needs after treatment.

### 4.3 Clinical implications

Clinicians can use these results to identify in a timely manner those women with breast cancer who are at risk of developing higher levels of unmet needs after treatment. Distress was found to be the most relevant risk factor across need domains. Additionally, older women with breast cancer, with one or more comorbid disorders, who have had chemotherapy, or who had a mastectomy may have more specific needs. Higher age was associated with higher health system‐related and informational needs. Comorbidity was associated with higher physical and daily living needs. Having had chemotherapy and a mastectomy were associated with higher sexuality needs and breast cancer‐specific issues, respectively.

Taking into consideration the growing number of breast cancer patients and staff shortages, this raises the question how patients' needs can be adequately met, and perhaps even prevented. Based on our results and the literature, distress should be targeted as one of the most significant and modifiable risk factors of unmet needs after treatment. One possible cost‐effective approach would be to address distress by use of a stepped care program, that is, watchful waiting, followed by guided self‐help, problem‐solving therapy, and psychotherapy or medication, if needed. Research shows that only a minority of patients will need the more resource‐intensive practices.^40^

Furthermore, the number of online self‐management tools that target specific needs or distress is steadily growing. However, most of the promising, evidence‐based, tools are not structurally updated and implemented in practice because of lack of funding after development. Also, potential users do not know which tools are available, and how to evaluate these tools in terms of quality, reliability, and privacy. Therefore, based on our study results, we also strongly encourage endeavors to successfully implement and disseminate such low resource, technology‐based aids among patients and clinicians.

## CONFLICT OF INTEREST

No financial or other conflictual relationships are disclosed that preclude publication of this manuscript.

## Data Availability

The data that support the findings of this study are available from the corresponding author upon reasonable request.
